# Effects of the angle of head‐down tilt on dynamic cerebral autoregulation during combined exposure to cephalad fluid shift and mild hypercapnia

**DOI:** 10.1113/EP091807

**Published:** 2024-09-04

**Authors:** Tomokazu Kato, Yojiro Ogawa, Ken‐ichi Iwasaki

**Affiliations:** ^1^ Department of Social Medicine, Division of Hygiene Nihon University School of Medicine Itabashi Tokyo Japan

**Keywords:** body fluid shift, cerebral autoregulation, hypercapnia

## Abstract

Astronauts experience combined exposure to a cephalad fluid shift and mild hypercapnia during space missions, potentially contributing to health problems. Such combined exposure may weaken dynamic cerebral autoregulation. The magnitude of cephalad fluid shift varies between individuals, and dynamic cerebral autoregulation may be affected more by greater cephalad fluid shift during combined exposure. We evaluated the dose‐dependent effects of head‐down tilt (HDT) on dynamic cerebral autoregulation during acute combined exposure to HDT and 3% CO_2_ inhalation. Twenty healthy participants were randomly exposed to three angles of HDT (−5°HDT+CO_2_, −15°HDT+CO_2_ and −30°HDT+CO_2_). After 15 min of rest, participants inhaled room air for 10 min in a horizontal body position, then inhaled 3% CO_2_ for 10 min under HDT. The last 6 min of data were used for analysis in each stage. Arterial pressure waveforms were obtained using finger blood pressure, and blood velocity waveforms in the middle cerebral artery were obtained using transcranial Doppler ultrasonography. Dynamic cerebral autoregulation was evaluated by transfer function analysis between waveforms. Statistical analysis was performed by two‐way repeated‐measures analysis of variance. The index of transfer function gain in the low‐frequency range increased significantly with −15°HDT+CO_2_ and −30°HDT+CO_2_, but no changes were seen with −5°HDT+CO_2_. Phase in the low‐frequency range decreased significantly with all three protocols. These results of significant changes in indexes of both gain and phase during combined exposure to steep HDT (−15° to −30°) and 3% CO_2_ inhalation suggest weakened dynamic cerebral autoregulation with the combination of moderate cephalad fluid shift and mild hypercapnia.

## Abstract (highlights)

- **What is the central question of this study?**
  Is dynamic cerebral autoregulation substantially affected by greater cephalad fluid shift during acute combined exposure to head‐down tilt (−5° to −30°) and 3% CO_2_ inhalation?
- **What is the main finding and its importance?**
  There were changes in both indexes of gain and phase during combined exposure to steep head‐down tilt (−15° to −30°) and 3% CO_2_ inhalation, suggesting weakened dynamic cerebral autoregulation by the combination of moderate cephalad fluid shift and mild hypercapnia.

## 1 INTRODUCTION

Manned missions to Mars will require closer attention to the health management of astronauts than International Space Station (ISS) missions due to the longer mission duration and greater isolation from Earth‐based medical support. These missions will therefore need to control various habitational and environmental factors such as gravity and ambient carbon dioxide (CO_2_) levels, which are likely to have effects on human health.

Astronauts aboard the ISS are exposed to microgravity and elevated ambient CO_2_ (Law et al., 2014) that would lead to 5–6 mmHg increases in end‐tidal CO_2_ ($ET_{CO_{2}}$) level (Hughson et al., 2016). Such conditions result in a combination of cephalad fluid shift and mild hypercapnia (Hughson et al., 2016). Previous studies have shown that a single exposure to 3% CO_2_ inhalation, which is approximately equivalent to the increase in $ET_{CO_{2}}$ (5–6 mmHg) observed on the ISS, did not attenuate dynamic cerebral autoregulation (Jeong et al., 2016; Kurazumi et al., 2017), whereas a single exposure to CO_2_ inhalation exceeding 5% did attenuate cerebral autoregulation (Aaslid et al., 1989; Jeong et al., 2016; Zhang et al., 1998). Previous studies have also shown that an acute single exposure to −10° to −30° head‐down tilt (HDT) does not affect dynamic cerebral autoregulation (Cooke et al., 2003; Kato et al., 2022; Kurazumi et al., 2017). However, a combination of −10° HDT and 3% CO_2_ inhalation attenuates dynamic cerebral autoregulation (Kurazumi et al., 2017). Health problems involving the brain and eyes, such as spaceflight‐associated neuro‐ocular syndrome (SANS) during long‐duration spaceflight (Mader et al., 2011) or space headache (Law et al., 2014) may thus be associated with attenuated dynamic cerebral autoregulation induced by the combined exposure to cephalad fluid shift and mild hypercapnia.

The degree of cephalad fluid shift seems to vary individually between astronauts. Our previous study showed that symptoms of cephalad fluid shift such as facial oedema and nasal congestion still persisted at 4 months in the ISS in 3 of 11 astronauts, whereas the remaining astronauts showed no apparent symptoms at that time (Iwasaki et al., 2021). Further, manned missions to the Moon or Mars will involve exposure to wider ranges of gravitational environments, potentially leading to more variety in levels of cephalad fluid shift. Although −6° to −10° HDT has generally been used to simulate cephalad fluid shift during exposure to microgravity in the field of space medicine (Kermorgant et al., 2020; Nicogossian et al., 2016), several levels of HDT may need to be investigated in ground‐based studies for SANS (Marshall‐Goebel et al., 2016). Using a shallow angle of HDT (e.g., −5°) to simulate mild cephalad fluid shift and steep angles of HDT (e.g., −15° to −30°) to simulate moderate cephalad fluid shift could be beneficial to account for individual variety in symptoms. Dynamic cerebral autoregulation may be affected more by greater cephalad fluid shift during combined exposure to cephalad fluid shift and mild hypercapnia. However, the dose‐dependence of effects from cephalad fluid shift on dynamic cerebral autoregulation during such combined exposures have not yet been clarified.

We therefore evaluated the dose‐dependence of effects from HDT (−5°, −15° and −30°) on dynamic cerebral autoregulation during acute combined exposure to HDT and 3% CO_2_ inhalation.

## 2 METHODS

### 2.1 Participants

Participants comprised 20 healthy volunteers (18 men, two women). Mean ± standard deviation (SD) age was 24 ± 1 years, mean height was 170 ± 8 cm and mean weight was 63 ± 9 kg. The study protocol was approved by the institutional review board at Nihon University School of Medicine (approval no. P20‐06‐2) and was registered to the University Hospital Medical Information Network clinical trial registry (ID: UMIN000044981). All procedures adhered to the tenets of the *Declaration of Helsinki*. All participants provided written informed consent as well as a medical history regarding cardiovascular health and were screened based on physical examinations including electrocardiography (ECG) and arterial blood pressure measurements. Participants were excluded if signals of blood velocity in the right middle cerebral artery (MCA) could not be obtained on transcranial Doppler ultrasonography (TCD) or if the individual had any history of major medical illness. All experiments were performed ≥2 h after a meal. Participants were asked to refrain from exercising heavily or consuming caffeinated or alcoholic beverages for at least 24 h before the experiments. All participants were familiarized with the measurement techniques and experimental conditions before the first data collection experiment.

### 2.2 Instrumentation

All experiments were performed in an environmentally controlled laboratory with an ambient temperature of 20−25°C and environmental CO_2_ <1000 ppm. Participants lay supine on an electrical tilting bed (Minato Medical Science, Osaka, Japan), initially placed in the horizontal position. Three‐lead ECG for heart rate (HR) and pulse oximetry for arterial oxygen saturation ($S_{pO_{2}}$) were applied (Lifescope BSM‐3800; Nihon Kohden, Tokyo, Japan). A vented mask was applied and connected via a Y‐connector to two Douglas bags, one filled with room air and the other filled with 3% CO_2_ (21%O_2_, 76%N_2_). Capnograms were obtained based on a side‐stream system using the external CO_2_ pressure device via an oral–nasal cannula with a sampling microtube (MicroPod; Oridion Medical, Jerusalem, Israel). Inspiratory CO_2_ pressure, respiratory rate and $ET_{CO_{2}}$ as detected from the capnogram were displayed on a monitor (Lifescope BSM‐3800; Nihon Kohden). Arterial pressure waveforms at the heart level were continuously measured from the right middle finger using a volume clamping method with photoplethysmography as part of the feedback loop used to maintain clamping (Finometer MIDI; Finapres Medical Systems, Amsterdam, the Netherlands) and a height‐correction sensor was placed on the right upper arm at heart level (xiphoid process level along the anterior axillary line). Intermittent arterial pressure was measured using the oscillometric method with a cuff sphygmomanometer placed over the left brachial artery at heart level before each data collection session (Lifescope BSM‐3800). Blood velocity waveforms in the MCA were obtained continuously by TCD (EZ‐Dop; Compumedics Germany GmbH, Sipplingen, Germany) at a depth of 50−60 mm using a 2 MHz probe, by the same experienced physician. The reproducibility of blood velocity in the MCA as measured by TCD is known to be good when performed by an experienced sonographer paying careful attention to probe placement. In the present study, the coefficient of variation in blood velocity in the MCA in the supine resting condition before HDT was 7.0%, as achieved using the following procedure for probe fixation. The probe was fixed individually at the same position and at a constant angle using a customized moulded probe holder made by the same experienced physician using an earplug and dental impression material to fit the facial bone and ear structure of each participant (Giller & Giller, 1997; Iwasaki et al., 2021). The same depth, power and sample volume for each participant were used for all three angles of HDT. Waveforms of finger arterial pressure at heart level, blood velocity in the MCA and ECG were recorded at a sampling rate of 1 kHz using commercial software (Notocord‐hem3.3; Notocord, Paris, France) throughout the study.

### 2.3 Experimental protocol

**FIGURE 1:**
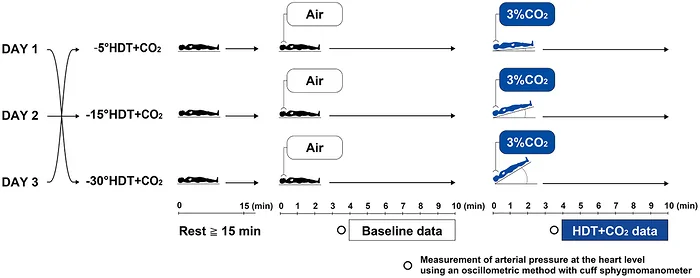
Experimental protocol. Participants were exposed to the three different angles of HDT during 3% CO_2_ inhalation (−5°HDT+CO_2_, −15°HDT+CO_2_ and −30°HDT+CO_2_) in random order on separate days. Following 15 min of rest on the tilting bed in a supine position, the participant inhaled the room air in one of the Douglas bags for 10 min via the vented mask in the horizontal body position. Data from the last 6 min of this period were used as baseline data. Tilt angle of the tilting bed was then changed to a −5°, −15° or −30° HDT position. At the same time as the HDT position was reached, inhalation of 3% CO_2_ from the other Douglas bag was started, continuing for 10 min in the HDT position. Data from the last 6 min were used as HDT+CO_2_ data. Intermittent arterial pressure was measured using the oscillometric method with a cuff sphygmomanometer before each data collection session.

Graphical representations of the experimental protocol are shown in Figure 1. Participants were exposed to the three different angles of HDT (−5°, −15°, and −30°) during 3% CO_2_ inhalation (−5°HDT+CO_2_, −15°HDT+CO_2_, and −30°HDT+CO_2_) in random order on separate days. Following 15 min of rest on the tilting bed in a supine position, the participant inhaled the room air in one of the Douglas bags for 10 min via the vented mask in the horizontal body position. Data from the last 6 min of this period (i.e., the 4th−10th min) were used as baseline data. Tilt angle of the tilting bed was then changed to a −5°, −15° or −30° HDT position. At the same time as the HDT position was reached, inhalation of 3% CO_2_ from the other Douglas bag was started, continuing for 10 min in the HDT position. Data from the last 6 min (4th−10th min) were used as HDT+CO_2_ data. The experiment was terminated if the participant reported symptoms of severe headache, nausea or vertigo.

### 2.4 Data analysis

#### 2.4.1 Steady‐state haemodynamics and respiratory conditions

Indices of steady‐state mean blood velocity in the MCA (MCAV) and HR were obtained by averaging recorded waveforms for the 6‐min data collection periods (4th−10th min) as steady‐state parameters. Mean arterial pressure at the heart level (MAP_Heart_) was obtained from the cuff sphygmomanometer immediately before beginning each data collection. To determine mean arterial pressure at brain level (MAP_Brain_), the difference in hydrostatic pressure between the heart level (level of the xiphoid process) and MCA level (level of the external acoustic meatus) was added to MAP_Heart_.

The difference in hydrostatic pressure (Pa) was calculated using the following formula:

$$\begin{array}{ccc} \mathrm{Hydrostatic}\;\mathrm{pressure}\;\mathrm{difference}\left(\mathrm{Pa}\right) & = & d\left(m\right)\times g\left(m/s^{2}\right)\\ & & \times \;\rho_{\mathrm{Blood}}\left(\mathrm{kg}/m^{3}\right) \end{array}$$

where d is the manually measured distance between the levels of the heart and MCA, *g* is gravitational acceleration (9.81 m/s^2^), $\rho_{\mathrm{Blood}}$ is the density of blood at 37°C as calculated from multiplying the specific gravity of whole blood at 37°C (1.06) and the density of water at 37°C (993 kg/m^3^) (Trudnowski & Rico, 1974). Units of hydrostatic pressure were then converted from pascals to millimetres of mercury (the standard unit of blood pressure) using the following formula:

$$\begin{array}{ccc} & & \mathrm{Hydrostatic}\;\mathrm{pressure}\;\mathrm{difference}\;\left(\mathrm{mmHg}\right)\\ & & \;=\;\frac{\mathrm{Hydrostatic}\;\mathrm{pressure}\;\mathrm{difference}\;\left(\mathrm{Pa}\right)}{0.001\;\left(m\right)\times \rho_{\mathrm{Hg}}\;\left(\mathrm{kg}/m^{3}\right)\times g\;\left(m/s^{2}\right)} \end{array}$$

where $\rho_{\mathrm{Hg}}$ is the density of mercury at 37°C as calculated from multiplying the specific gravity of mercury at 37°C (13.6) and the density of water at 37°C (993 kg/m^3^). The hydrostatic pressure difference (mmHg) between the levels of the heart and MCA was thus calculated from *d* using the following formula:

$$\mathrm{Hydrostatic}\;\mathrm{pressure}\;\mathrm{difference}\;\left(\mathrm{mmHg}\right)=d\;\left(m\right)\times 1.06/13.6\times 1000$$

Respiratory rate, $S_{pO_{2}}$ and $ET_{CO_{2}}$ were manually recorded every minute and used to calculate averaged data for the 6‐min data collection periods.

#### 2.4.2 Spectral and transfer function analyses

Spectral and transfer function analyses were based on Cerebrovascular Research Network (CARNet)‐recommended algorithms (Claassen et al., 2016; Panerai et al., 2023), to allow comparison with other studies. These data analyses were performed using DADiSP software (DSP Development, Cambridge, MA, USA). Briefly, beat‐to‐beat mean arterial pressure at the heart level and mean blood velocity in the MCA were obtained from recorded waveforms for 6‐min data collection periods (4th−10th min) by integrating signals within each cardiac cycle using PC‐based software (Notocord‐hem3.3). Beat‐to‐beat mean arterial pressure and mean blood velocity in the MCA were linearly interpolated and resampled at 4 Hz for spectral and transfer function analyses. The time series of data were first subtracted from the mean values of the data collection period. Spectra for mean arterial pressure variability and mean blood velocity variability in the MCA were obtained by fast Fourier transform. Transfer functions between these two variabilities were calculated by the cross‐spectrum method to assess the dynamic relationship between arterial pressure and blood velocity in the MCA. The fast Fourier transform and transfer function analyses were performed using a Hamming window on 512‐point segments with 50% overlap. This process resulted in five segments of data recording over 6 min (the last segment comprised approximately 416 datapoints). Spectral smoothing was performed using a triangular moving‐average window with coefficients (¼, ½, ¼). The spectral power of mean arterial pressure variability (MAPv) and mean blood velocity variability in the MCA (MCAVv) were then calculated by integrating each variability in the very low frequency (VLF; 0.02−0.07 Hz), low frequency (LF; 0.07−0.20 Hz) and high frequency (HF; 0.20−0.35 Hz) ranges. Values of transfer function gain, phase and coherence were averaged in the VLF (0.02−0.07 Hz), LF (0.07−0.20 Hz) and HF (0.20−0.35 Hz) ranges. These ranges were specifically based on the frequency‐dependent property of dynamic cerebral autoregulation as previously proposed by transfer function analysis (Giller, 1990; Zhang et al., 1998). To correct phase wrap‐around for calculating phase values in the VLF and LF ranges, negative values below 0.1 Hz were removed by visually inspecting the phase plots. In addition, for two participants whose phase‐LF at −30°HDT+CO_2_ were outliers as determined by Q‐Q plot, negative values below 0.2 Hz were removed. Transfer function gain is represented as the absolute value (Gain) and the relative value (NGain) normalized with MAP_Brain_ and MCAV. If coherence does not exceed the critical value, data for transfer function gain and phase should be excluded from analysis, as the results can be expected to be unreliable (Claassen et al., 2016; Panerai et al., 2023). We therefore excluded data on transfer function gain and phase from our analysis when coherence did not exceed 0.34, as the critical value for coherence using five windows at a 95% confidence interval (Claassen et al., 2016; Panerai et al., 2023).

#### 2.4.3 Statistical analysis

Data are presented as means ± SD. All statistical analyses were performed using SigmaPlot software (version 14.5; Systat Software, Chicago, IL, USA). Normal distributions of data were confirmed using the Shapiro–Wilk test. Physiological variables were compared using two‐way repeated‐measures analysis of variance (ANOVA) with Stage (baseline and HDT+CO_2_) × Angle (−5°, −15°, and −30°), followed by a Bonferroni *t* test for multiple comparisons. Values of *P *< 0.05 were considered statistically significant. If data showed a non‐normal distribution, logarithmic transformation was performed before ANOVA. If variables did not follow a normal distribution even after logarithmic transformation, baseline and HDT+CO_2_ data were compared using the Wilcoxon signed rank test, with Bonferroni correction for each angle of the protocol.

## 3 RESULTS

All participants completed all data collections. No participants complained of severe headache or nausea during the experiments.

### 3.1 Steady‐state haemodynamics and respiratory condition

**FIGURE 2:**
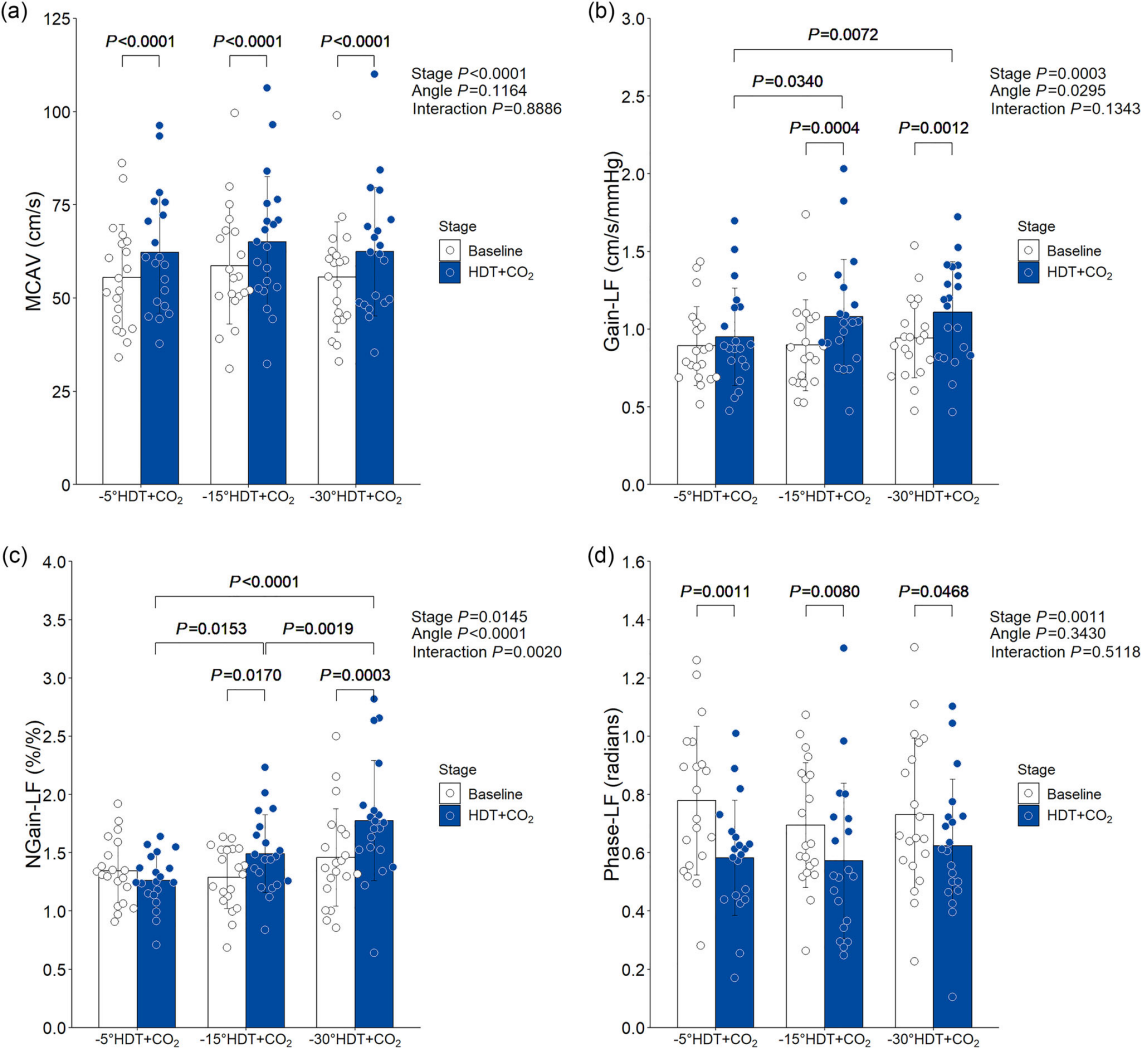
Group averages and individual changes in 6‐min average of mean blood velocity in the middle cerebral artery (MCAV) (a), transfer function gain in the low‐frequency range (Gain‐LF) (b), normalized transfer function gain in the low‐frequency range (NGain‐LF) (c), and phase in the low‐frequency range (Phase‐LF) (d). Grey bars: baseline data (supine + air inhalation); blue bars: HDT+CO_2_ data (head‐down tilt + 3% carbon dioxide inhalation). All variables were compared using two‐way repeated‐measures analysis of variance with Stage (baseline and HDT+CO_2_) × Angle (−5°, −15° and −30°), followed by a Bonferroni *t* test for multiple comparisons. A *P*‐value <0.05 was considered statistically significant.

In MAP_Brain_, a significant interaction (*P *< 0.0001), main effects of stage (*P *< 0.0001) and angle (*P *< 0.0001) were observed. MAP_Brain_ significantly increased with −15°HDT+CO_2_ and −30°HDT+CO_2_. MAP_Brain_ was significantly higher during −15°HDT+CO_2_ than during −5°HDT+CO_2_. MAP_Brain_ during −30°HDT+CO_2_ was significantly higher than during −5°HDT+CO_2_ or −15°HDT+CO_2_. No significant changes in MAP_Heart_ were identified. In MCAV (Figure 2a), a significant main effect of Stage (*P *< 0.0001) was observed. MCAV increased significantly to a similar extent under all three protocols (−5°HDT+CO_2_: +12%; −15°HDT+CO_2_: +10%; −30°HDT+CO_2_: +12%). No significant changes in HR were identified. In $S_{pO_{2}}$, a significant main effect of stage (*P *< 0.0001) was observed. $S_{pO_{2}}$ increased significantly with all three angles of HDT (Table 1).

**TABLE 1:** Steady‐state haemodynamics and respiratory condition

	−5°HDT+CO_2_ (*n* = 20)		−15°HDT+CO_2_ (*n* = 20)		−30°HDT+CO_2_ (*n* = 20)		*P* (ANOVA)		
	Baseline	HDT+CO_2_	Baseline	HDT+CO_2_	Baseline	HDT+CO_2_	Stage	Angle	Interaction
MAP_Brain_ (mmHg)	82 ± 6	82 ± 8	83 ± 6	89 ± 8^*^, ^†††^	83 ± 6	97 ± 7^*^, ^†††^, ^‡‡‡^	**<0.0001**	**<0.0001**	**<0.0001**
MAP_Heart_ (mmHg)	82 ± 6	81 ± 8	83 ± 6	82 ± 8	83 ± 6	83 ± 7	0.2787	0.3395	0.5792
MCAV (cm/s)	55 ± 14	62 ± 16^*^	58 ± 15	65 ± 17^*^	55 ± 14	62 ± 17^*^	**0.0001**	0.1164	0.8886
HR (beat/min)	67 ± 4	66 ± 4	67 ± 8	67 ± 6	66 ± 6	66 ± 5	0.1551	0.6196	0.9289
$S_{pO_{2}}$ (%)	97 ± 0	98 ± 0^*^	97 ± 1	98 ± 0^*^	97 ± 1	98 ± 0^*^	**<0.0001**	0.1344	0.9607
Resp‐R (breath/min)	13 ± 3	15 ± 4^*^	14 ± 2	15 ± 2	13 ± 3	14 ± 4	Wilcoxon signed rank test		
$ET_{CO_{2}}$ (Torr)	40 ± 2	45 ± 2^*^	40 ± 5	45 ± 3^*^	40 ± 3	44 ± 2^*^	Wilcoxon signed rank test		

*Note*: Values represent means ± standard deviation. For Resp‐R and $ET_{CO_{2}}$, the baseline and HDT+CO_2_ data were compared using Wilcoxon signed rank test with Bonferroni correction because the normality tests were failed.

* *P* < 0.05 (compared with each baseline).

^†††^ *P* < 0.001 (compared with intervention of –5°HDT+CO_2_).

^‡‡‡^ *P* < 0.001 (compared with intervention of –15°HDT+CO_2_). Abbreviations: $ET_{CO_{2}}$, end‐tidal carbon dioxide pressure; HDT, head‐down tilt; HR, heart rate; MAP_Brain_, mean arterial pressure at the brain level; MAP_Heart_, mean arterial pressure at the heart level; MCAV, mean blood velocity in the middle cerebral artery; Resp‐R, respiratory rate; $S_{pO_{2}}$, arterial oxygen saturation. Bold values show statistically significant (*P* < 0.05).

The Wilcoxon signed rank test with Bonferroni correction showed that respiratory rate increased significantly with −5°HDT+CO_2_. $ET_{CO_{2}}$ increased significantly with all three angles of HDT+CO_2_.

### 3.2 Spectral and transfer function analyses

**FIGURE 3:**
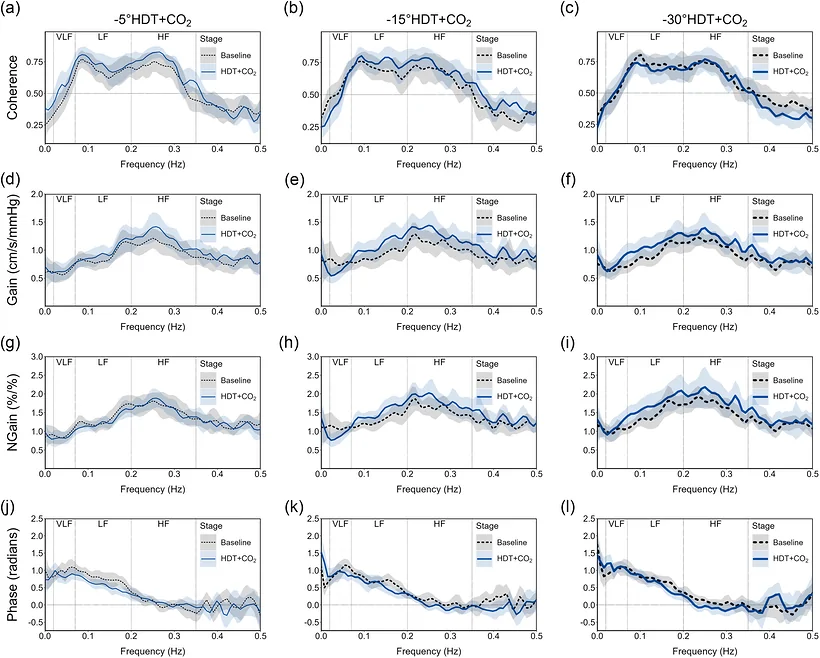
Group‐averaged transfer function analysis between mean arterial pressure (MAP) and mean blood velocity in the middle cerebral artery (MCAV) in three head‐down tilt (HDT) protocols (−5°HDT+CO_2_, −15°HDT+CO_2_, −30°HDT+CO_2_). (a–c) Coherence function (Coherence). (d–f) Transfer function gain (Gain). (g–i) Normalized transfer function gain between arterial pressure variability and blood velocity variability in the middle cerebral artery (NGain). (j–l) Phase relationship between arterial pressure variability and blood velocity variability in the middle cerebral artery (Phase). VLF, very low‐frequency range (0.02–0.07 Hz); LF, low‐frequency range (0.07–0.20 Hz); HF, high‐frequency range (0.20–0.35 Hz). Dashed line, group‐averaged baseline data; continuous line, group‐averaged HDT+CO_2_ data; grey shaded area, 95% confidence interval for baseline data; blue shaded area, 95% confidence interval for HDT+CO_2_ data.

The most pronounced changes were observed in the LF range. In the LF range of the present study, even the participant with the lowest coherence value exhibited a value exceeding 0.34 (Supporting information, Supplementary file 1). Transfer function gain and phase in the LF range were thus considered as acceptable estimates for all 20 participants. In Gain‐LF (Figure 2b), significant main effects of stage (*P *= 0.0003) and angle (*P *= 0.0295) were observed. Gain‐LF increased significantly with −15°HDT+CO_2_ and −30°HDT+CO_2_, but did not change with −5°HDT+CO_2_. Gain‐LF was significantly higher during −15°HDT+CO_2_ and −30°HDT+CO_2_ than during −5°HDT+CO_2_. In NGain‐LF (Figure 2c), a significant interaction (*P *= 0.0020), main effects of stage (*P *= 0.0145) and angle (*P *< 0.0001) were observed. NGain‐LF increased significantly with −15°HDT+CO_2_ and −30°HDT+CO_2_, although NGain‐LF did not change with −5°HDT+CO_2_. NGain‐LF was significantly higher during −15°HDT+CO_2_ than during −5°HDT+CO_2_. NGain‐LF was significantly higher during −30°HDT+CO_2_ than during −5°HDT+CO_2_ or −15°HDT+CO_2_. In Phase‐LF (Figure 2d), a significant main effect of stage was observed (*P *= 0.0011). Phase‐LF decreased significantly with all three angles of HDT+CO_2_. No significant changes in Coherence‐LF were identified. In LF‐MAPv, a significant interaction (*P *= 0.0015) and main effect of stage (*P *= 0.0082) were observed. LF‐MAPv decreased significantly with −15°HDT+CO_2_ and −30°HDT+CO_2_, but no significant change in LF‐MCAVv was observed (Table 2 and Figure 3).

**TABLE 2:** Spectral and transfer function analyses

	−5°HDT+CO_2_		−15°HDT+CO_2_		−30°HDT+CO_2_		*P* (ANOVA)		
	Baseline	HDT+CO_2_	Baseline	HDT+CO_2_	Baseline	HDT+CO_2_	Stage	Angle	Interaction
VLF (0.02–0.07 Hz), *n* = 9									
VLF‐MAPv (mmHg^2^)	5.84 ± 3.51	8.71 ± 5.99	7.31 ± 5.80	4.96 ± 3.51	8.18 ± 8.06	4.46 ± 2.21^*^	0.3994	0.7537	**0.0169**
VLF‐MCAVv (cm^2^/s^2^)	5.17 ± 3.74	5.55 ± 3.18	6.61 ± 4.63	4.79 ± 3.91	6.96 ± 9.73	5.35 ± 5.15	0.2162	0.9143	0.3826
Gain‐VLF (cm/s/mmHg)	0.70 ± 0.30	0.71 ± 0.36	0.79 ± 0.33	0.68 ± 0.24	0.67 ± 0.26	0.82 ± 0.29	0.5628	0.8193	0.0822
NGain‐VLF (%/%)	1.07 ± 0.38	1.00 ± 0.44	1.12 ± 0.25	1.02 ± 0.30	1.02 ± 0.36	1.31 ± 0.37^*^, ^†^, ^‡^	0.3858	0.3052	**0.0394**
Phase‐VLF (radians)	1.02 ± 0.36	0.89 ± 0.42	1.07 ± 0.41	0.92 ± 0.28	0.96 ± 0.26	1.17 ± 0.24	0.7174	0.7004	0.0664
Coherence‐VLF	0.56 ± 0.13	0.65 ± 0.14^*^	0.59 ± 0.17	0.56 ± 0.18	0.61 ± 0.15	0.68 ± 0.17	**0.0303**	0.2854	0.2036
LF (0.07–0.20 Hz), *n* = 20									
LF‐MAPv (mmHg^2^)	3.04 ± 1.86	3.03 ± 1.91	3.35 ± 2.71	2.34 ± 1.66^*^	3.24 ± 2.45	2.04 ± 1.61^*^	**0.0082**	0.6657	**0.0015**
LF‐MCAVv (cm^2^/s^2^)	2.82 ± 2.68	3.06 ± 3.06	3.10 ± 2.81	2.78 ± 1.95	3.02 ± 2.52	2.64 ± 2.32	0.4843	0.9756	0.2367
Gain‐LF (cm/s/mmHg)	0.89 ± 0.25	0.95 ± 0.31	0.89 ± 0.29	1.08 ± 0.36^*^, ^†^	0.94 ± 0.25	1.10 ± 0.32^*^, ^†^	**0.0003**	**0.0295**	0.1343
NGain‐LF (%/%)	1.34 ± 0.27	1.26 ± 0.23	1.29 ± 0.26	1.49 ± 0.33^*^, ^†^	1.46 ± 0.41	1.77 ± 0.51^*^, ^†^, ^‡^	**0.0145**	**<0.0001**	**0.0020**
Phase‐LF (radians)	0.77 ± 0.25	0.58 ± 0.19^*^	0.69 ± 0.21	0.57 ± 0.26^*^	0.73 ± 0.26	0.62 ± 0.22^*^	**0.0011**	0.3430	0.5118
Coherence‐LF	0.69 ± 0.11	0.73 ± 0.13	0.69 ± 0.13	0.75 ± 0.15	0.72 ± 0.11	0.69 ± 0.17	0.2785	0.7946	0.0965
HF (0.20–0.35 Hz), *n* = 19									
HF‐MAPv (mmHg^2^)	0.19 ± 0.11	0.25 ± 0.17	0.25 ± 0.21	0.37 ± 0.43	0.22 ± 0.19	0.31 ± 0.40	**0.0121**	0.8022	0.9318
HF‐MCAVv (cm^2^/s^2^)	0.30 ± 0.19	0.50 ± 0.35^*^	0.39 ± 0.23	0.62 ± 0.53^*^	0.33 ± 0.24	0.59 ± 0.47^*^	Wilcoxon signed rank test		
Gain‐HF (cm/s/mmHg)	1.07 ± 0.30	1.21 ± 0.35^*^	1.13 ± 0.36	1.27 ± 0.35^*^	1.07 ± 0.25	1.21 ± 0.31^*^	**<0.0001**	0.5575	0.9955
NGain‐HF (%/%)	1.64 ± 0.44	1.63 ± 0.35	1.64 ± 0.35	1.80 ± 0.48	1.66 ± 0.38	1.97 ± 0.59^*^	**0.0017**	0.2317	0.0745
Phase‐HF (radians)	0.04 ± 0.19	0.09 ± 0.18	0.09 ± 0.13	0.00 ± 0.16^*^	0.10 ± 0.24	−0.02 ± 0.21^*^	Wilcoxon signed rank test		
Coherence‐HF	0.67 ± 0.10	0.73 ± 0.07^*^	0.68 ± 0.15	0.72 ± 0.13	0.66 ± 0.10	0.67 ± 0.13	**0.0028**	0.2848	0.2413

*Note*: Values represent means ± standard deviation. For HF‐MCAV and Phase‐HF, the baseline and HDT+CO_2_ data were compared using Wilcoxon signed rank test with Bonferroni correction because the normality tests were failed.

* *P* < 0.05 (compared with each baseline).

^†^ *P* < 0.05 (compared with stage of –5°HDT+CO_2_).

^‡^ *P* < 0.05 (compared with stage of –15°HDT+CO_2_).

Abbreviations: HDT, head‐down tilt; HF, high frequency range (0.20–0.35 Hz); Gain, transfer function gain; LF, low frequency range (0.07–0.20 Hz); MAPv, mean arterial pressure variability at the heart level; MCAVv, mean cerebral blood velocity variability in the middle cerebral artery; NGain, normalized transfer function gain; VLF, very low frequency range (0.02–0.07 Hz). Bold values show statistically significant (*P* < 0.05).

Eleven participants were excluded from analysis in the VLF range because of Coherence‐VLF values below 0.34. No significant changes in Gain‐VLF were observed. In NGain‐VLF, a significant interaction (*P *= 0.0394) was observed. NGain‐VLF increased significantly with −30°HDT+CO_2_. NGain‐VLF was significantly higher during −30°HDT+CO_2_ than during −5°HDT+CO_2_ or −15°HDT+CO_2_. No significant changes in Phase‐VLF were identified. A significant main effect of stage (*P *= 0.0303) was observed in Coherence‐VLF. Coherence‐VLF increased significantly with −5°HDT+CO_2_. In VLF‐MAPv, a significant interaction (*P *= 0.0169) was observed. VLF‐MAPv decreased significantly with −30°HDT+CO_2_. No significant changes in VLF‐MCAVv were identified.

One participant was excluded from analysis in the HF range because of Coherence‐HF values below 0.34. Gain‐HF showed a significant main effect of stage (*P *< 0.0001). Gain‐HF increased significantly with all three angles of HDT+CO_2_. A significant main effect of stage was observed in NGain‐HF (*P *= 0.0017). NGain‐HF increased significantly with −30°HDT+CO_2_. The Wilcoxon signed‐rank test with Bonferroni correction showed that Phase‐HF decreased significantly with −15°HDT+CO_2_ and −30°HDT+CO_2_. In Coherence‐HF, a significant main effect of stage was observed (*P *= 0.0028). Coherence‐HF increased significantly with −5°HDT+CO_2_. Although a significant main effect of stage was observed in HF‐MAPv (*P *= 0.0121), multiple comparisons showed no significant changes. The Wilcoxon signed‐rank test with Bonferroni correction showed that HF‐MCAVv increased significantly with all three angles of HDT+CO_2_.

## 4 DISCUSSION

The main findings of the present study were as follows. First, although neither Gain‐LF nor NGain‐LF changed significantly during −5°HDT+CO_2_, both increased significantly during −15°HDT+CO_2_ and −30°HDT+CO_2_ compared with the respective baselines. Second, Phase‐LF decreased significantly during the combination of all three angles of HDT+CO_2_, although no significant differences in Phase‐LF were observed among the three protocols. These findings for changes in both indices of transfer function gain and phase during combined exposure to steep HDT (−15° to −30°) and 3% CO_2_ inhalation suggest weakened dynamic cerebral autoregulation under the combination of moderate or greater cephalad fluid shift and mild hypercapnia.

### 4.1 Dynamic cerebral autoregulation

Although slight differences in the magnitude of changes and the statistical results were observed between Gain‐LF and NGain‐LF in the present study, both Gain‐LF and NGain‐LF increased significantly during −15°HDT+CO_2_ and −30°HDT+CO_2_, but not during −5°HDT+CO_2_. Transfer function gain indicates magnitude relationships between arterial pressure oscillations and cerebral blood flow fluctuations. The present results for Gain‐LF and NGain‐LF together thus suggest that the magnitude relationship between arterial pressure oscillations and cerebral blood flow fluctuations may increase under combined exposure to steep HDT (−15° to −30°) and 3% CO_2_ inhalation. This implies that any given changes in arterial pressure lead to amplified changes in blood velocity in the MCA under these conditions. In addition, the present study showed that LF‐MCAVv did not decrease during −15°HDT+CO_2_ or −30°HDT+CO_2_, although LF‐MAPv decreased significantly during −15°HDT+CO_2_ and −30°HDT+CO_2_. These results indicate that cerebral blood flow fluctuations may show relative increases against arterial pressure oscillations during −15°HDT+CO_2_ and −30°HDT+CO_2_, also supporting the interpretations of an increased magnitude relationship between arterial pressure oscillations and cerebral blood flow fluctuations.

In contrast, regarding the significant decreases in Phase‐LF during all three angles of HDT, no marked differences were observed among the three protocols. Phase is determined from the time delay between arterial pressure oscillations and cerebral blood flow fluctuations (Panerai et al., 2023). The relationship in the LF range would change under combined exposure to HDT and 3% CO_2_ inhalation, regardless of the angle of HDT from −5° to −30°.

The results of transfer function gain and phase together indicate that combined exposure to moderate cephalad fluid shift (−15° to −30° HDT) and mild hypercapnia (3% CO_2_ inhalation) notably attenuates dynamic cerebral autoregulation. In addition, these findings, together with a previous study by our research group for −10°HDT+3% CO_2_ (Kurazumi et al., 2017), appear to suggest that such attenuations may be observed with HDT exceeding −10°.

The present study observed significant increases in Gain‐HF during all three angles of HDT. A previous study reported an increase in the power of R‐R interval variability in the HF range (HF_RRI_) as an index of respiratory sinus arrhythmia during HDT (Porta et al., 2015), possibly indicating increases in parasympathetic activity. The present study also observed significant increases in HF_RRI_ during three angles of HDT (Supporting information, Supplementary file 2), consistent with findings from the previous study. An increase in the mechanical effects of respiratory condition may also affect transfer function gain in respiratory frequency ranges. Increases in parasympathetic activity and/or mechanical effects of respiratory condition may therefore be the mechanisms responsible for the increase in Gain‐HF in the present study. Moreover, these two factors may also affect transfer function gain in the LF range in participants with respiratory rate below 12 breaths/min (0.20 Hz), as the respiratory frequency falls within the LF range (Supporting information, Supplementary file 3 and 4). These cases could be a confounding factor for the results of transfer function gain in the LF range.

### 4.2 Steady‐state cerebral blood flow

Previous studies have shown that 10 min of 3% CO_2_ inhalation increased mean MCAV by 14% (Kurazumi et al., 2017) and 10% (Jeong et al., 2016). Further, Kurazumi et al. found no significant differences in mean MCAV between supine and −10° HDT during 3% CO_2_ inhalation. The present investigation found similar increases in mean MCAV during all three angles of HDT (−5°HDT+CO_2_: +12%; −15°HDT+CO_2_: +10%; −30°HDT+CO_2_: +12%). As a result, HDT up to −30° may have no additional effects on the increases in steady‐state cerebral blood flow caused by mild hypercapnia with 3% CO_2_ inhalation.

### 4.3 Clinical implications

Individual variations have been reported in the symptoms of cephalad fluid shift, such as facial oedema and eye changes associated with SANS during exposure to microgravity environments (Iwasaki et al., 2021; Lee et al., 2018). Additionally, astronauts on manned missions to the Moon and Mars will be exposed to a wide range of gravity levels (ISS and Gateway: 0 G; Moon: 1/6 G; Mars: 3/8 G; Earth: 1 G), presumably resulting in varying degrees of cephalad fluid shift. In addition to such a variety in cephalad fluid shift, there is a risk of hypercapnia for astronauts on spacecraft and in space stations and bases. In recent clinical practice, robot‐assisted laparoscopic surgeries have been performed in the fields of gynecology and urology, using approximate −10° to −30° HDT to achieve better visualizations of target organs (Aggarwal et al., 2021; Ghomi et al., 2012). Steeper HDT in patients undergoing laparoscopic surgery is reportedly related to various complications, such as brain oedema and hemiparesis (Barr et al., 2014; Pandey et al., 2012). Dynamic cerebral autoregulation might be further attenuated in astronauts with intense body fluid shifts and in patients undergoing laparoscopic surgery under steep HDT, risking the induction of various health problems.

### 4.4 Limitations

Four major limitations should be considered in the present study. First, the evaluation of cerebral blood flow by TCD is based on the assumption that MCA diameter remains constant. Verbree et al. confirmed an absence of significant changes in MCA diameter during hypercapnia with a 7.5 mmHg increase in $ET_{CO_{2}}$ using 7 T ultrahigh‐resolution MRI (Verbree et al., 2014), but found a significant increase in MCA diameter with a 15 mmHg increase in $ET_{CO_{2}}$. Coverdale et al. likewise reported an increase in the cross‐sectional area of the MCA by the first minute during an approximately 10 mmHg increase in $ET_{CO_{2}}$ with 6% CO_2_ using 3 T MRI (Coverdale et al., 2014). Those studies indicated that MCA diameter may be relatively constant within a 7.5 mmHg increase in $ET_{CO_{2}}$. In the present study, the increases in $ET_{CO_{2}}$ induced by inhaling 3% CO_2_ were approximately 5 mmHg under all three protocols. However, we cannot exclude the possibility that MCA diameter changed during combined exposure to HDT and 3% CO_2_ inhalation.

**FIGURE 4:**
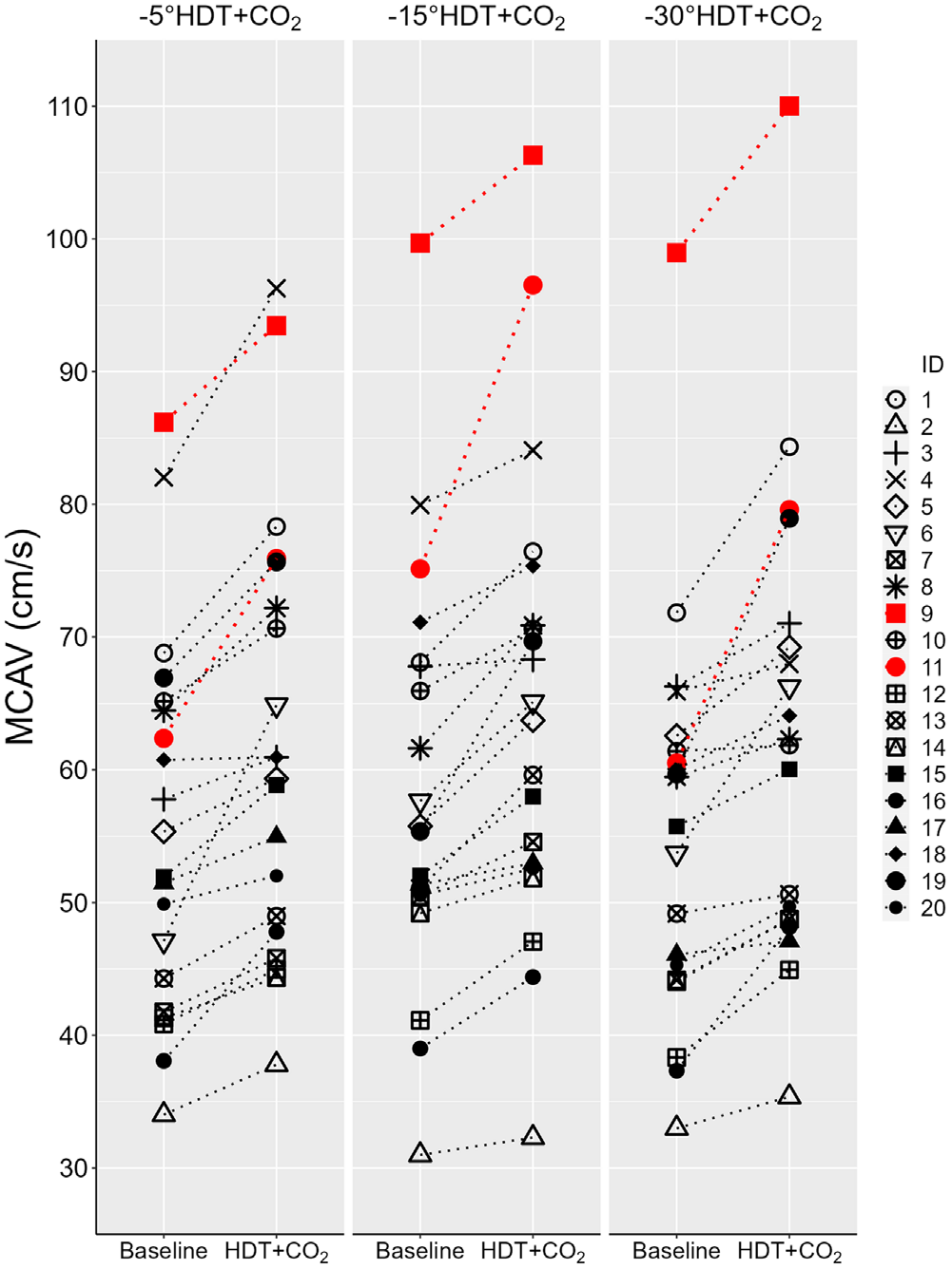
Individual changes in 6‐min average of mean blood velocity in the middle cerebral artery (MCAV) in three head‐down tilt (HDT) protocols (−5°HDT+CO_2_, −15°HDT+CO_2_, −30°HDT+CO_2_). Red symbols represent the data of the two female participants, while black symbols represent the data of male participants.

Second, we did not attempt to balance the sex ratio of participants during recruitment and only two women applied to participate in this study, resulting in a predominance of male participants. The results of this study might thus be more applicable to men than to the general human population. Higher cerebral blood flow and faster blood velocity in the MCA during quiet rest have been reported in women compared to men (Gur et al., 1982; Liu et al., 2012; Minhas et al., 2018; Wang et al., 2005; Xing et al., 2017). The present study also observed a slight tendency toward faster MCAV in the two female participants compared to male participants (Figure 4), consistent with previous studies. Larger increases in blood velocity in the MCA induced by CO_2_ inhalation have been also reported in women compared to men (Kastrup et al., 1998; Minhas et al., 2018). These sex differences in cerebral blood flow and CO_2_ reactivity could potentially lead to differential effects between men and women under combined exposure to HDT and 3% CO_2_ inhalation. However, the present study was unable to elucidate sex differences due to the small number of female participants. In addition, results of cerebral blood flow and CO_2_ reactivity may differ depending on the timing of the experiment and ages of the participants in terms of the menstrual cycle and sex hormone levels. $ET_{CO_{2}}$ is well‐known to tend to decrease in the luteal phase of the menstrual cycle due to an increase in ventilation stimulated by progesterone (Brodeur et al., 1986; Selvi et al., 2012; Skatrud et al., 1978). Therefore, cerebral vascular tone could be affected by changes in $ET_{CO_{2}}$ across the menstrual cycle, possibly affecting dynamic cerebral autoregulation. To clarify the effects of sex differences on dynamic cerebral autoregulation, future studies with sufficient numbers of female participants, data collection times controlling for the menstrual cycle and participants of various ages are needed.

Third, the present study did not include a protocol for single exposures to either HDT or 3% CO_2_ inhalation alone. Therefore, we can only rely on previous studies to infer the individual effects on dynamic cerebral autoregulation.

Fourth, in the VLF range, 11 of 20 participants showed coherence below 0.34, the critical value of coherence presented in the white paper (Claassen et al., 2016; Panerai et al., 2023). The results of transfer function gain and phase in the VLF range may thus be unreliable in the present study.

### 4.5 Conclusion

The present results showed that transfer function gains in the LF range increased under combined exposure to −15° to −30° HDT and 3% CO_2_ inhalation, but not during combined exposure to −5° HDT and 3% CO_2_ inhalation. In addition, phase in the LF range decreased regardless of the angle of HDT. Significant changes in both indexes of transfer function gain and phase were thus observed during steep HDT (−15° to −30°). In conclusion, combined exposure to moderate cephalad fluid shift and mild hypercapnia may weaken dynamic cerebral autoregulation.

## AUTHOR CONTRIBUTIONS

Tomokazu Kato helped with the design of the study, acquisition, analysis and interpretation of data, and drafting the manuscript. Yojiro Ogawa helped with the acquisition and interpretation of data and revision of the manuscript. Ken‐ichi Iwasaki helped with the conception and design of the study, acquisition, analysis and interpretation of data, and revision of the manuscript. All authors have read and approved the final version of this manuscript and agree to be accountable for all aspects of the work in ensuring that questions related to the accuracy or integrity of any part of the work are appropriately investigated and resolved. All persons designated as authors qualify for authorship, and all those who qualify for authorship are listed.

## CONFLICT OF INTEREST

The authors declare that no conflicts of interest exist.

## Supporting information

Supplementary figures and tables.

## Data Availability

All individual data supporting the results are presented in the manuscript figures. The datasets used and/or analysed in the present study are available from the corresponding author on reasonable request.
