# Supplementary material for: Effects of the angle of head‐down tilt on dynamic cerebral autoregulation during combined exposure to cephalad fluid shift and mild hypercapnia
Source: Exp Physiol. 2024 Sep 4:10.1113/EP091807. Online ahead of print. doi: 10.1113/EP091807 (PMC13394826; doi:10.1113/EP091807)
Supplement: Supplementary file 1 — Supplementary figures and tables. [file EPH-9999-0-s001.docx]

**Supporting Information**

**Supplementary file 1.** Individual changes in Coherence-LF in three head-down tilt (HDT) protocols (-5°HDT+CO_2_, -15°HDT+CO_2_, -30°HDT+CO_2_). Each symbol represents the Coherence-LF of an individual. The red dotted line indicates a coherence value of 0.34, the critical value of coherence with 5 windows using the 95% confidence interval (Claassen et al., 2016; Panerai et al., 2023). No participants with Coherence-LF below 0.34 were observed in the present study.


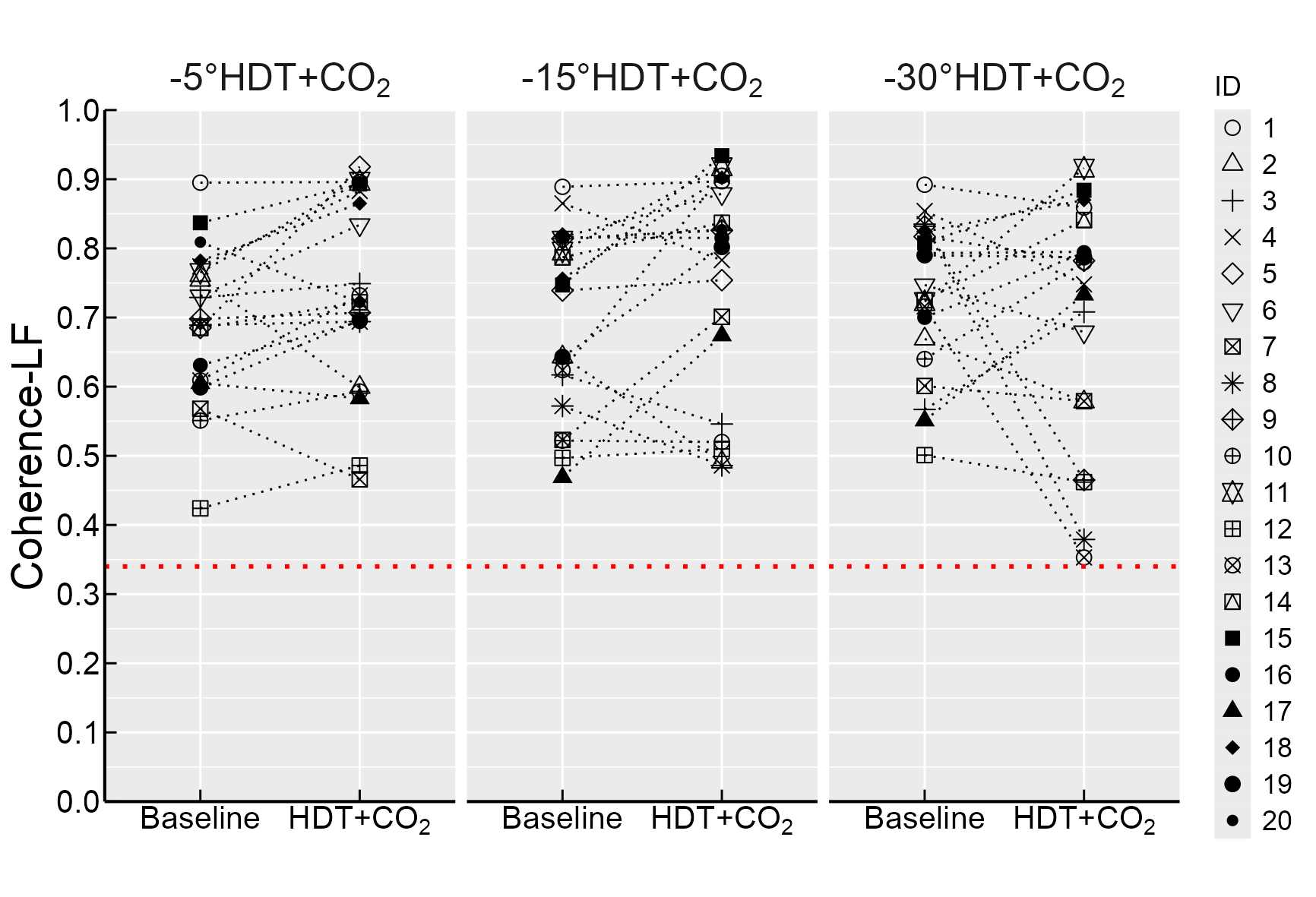


| **Supplementary file 2. Power of R-R interval variability in the high frequency range** | | | | | | | | | | | | | | | | | | | | | | | |  | |  | |  | |  | |  |
| --- | --- | --- | --- | --- | --- | --- | --- | --- | --- | --- | --- | --- | --- | --- | --- | --- | --- | --- | --- | --- | --- | --- | --- | --- | --- | --- | --- | --- | --- | --- | --- | --- |
|  | -5°HDT+CO_2_ (N=20) | | | | | |  | -15°HDT+CO_2_ (N=20) | | | | | | |  | | -30°HDT+CO_2_ (N=20) | | | | | | |  | | ANOVA | | | | | |  |
|  | Baseline | | | HDT+CO_2_ | | |  | Baseline | | | HDT+CO_2_ | | | |  | | Baseline | | | HDT+CO_2_ | | | |  | | Stage | | Angle | | Interaction | |  |
| HF_RRI_, ms^2^ | 578 | ± | 456 | 1301 | ± | 980* |  | 582 | ± | 306 | 1432 | ± | 848* |  | | 888 | | ± | 842 | 1653 | ± | 1420* |  | | ***P*<0.0001** | | ***P*=0.1020** | | ***P*=0.5412** | |  |  |
| Values represent mean ± standard deviation. HDT, head-down tilt; HF_RRI_, power of R–R interval variability in the high frequency range. **P*<0.05 (compared with each baseline). | | | | | | | | | | | | | | | | | | | | | | | | | | | | | | | |  |
|  |  |  |  |  |  |  |  |  |  |  |  |  |  |  |  |  |  |  |  |  |  |  |  |  |  |  |  |  |  |  |  |  |

**Supplementary file 3.** Individual changes in 6-min average respiratory rate in the three HDT protocols (-5°HDT+CO_2_, -15°HDT+CO_2_, -30°HDT+CO_2_). Each symbol represents the respiratory rate of an individual. The red dotted line indicates 12 breaths/min (0.20 Hz).


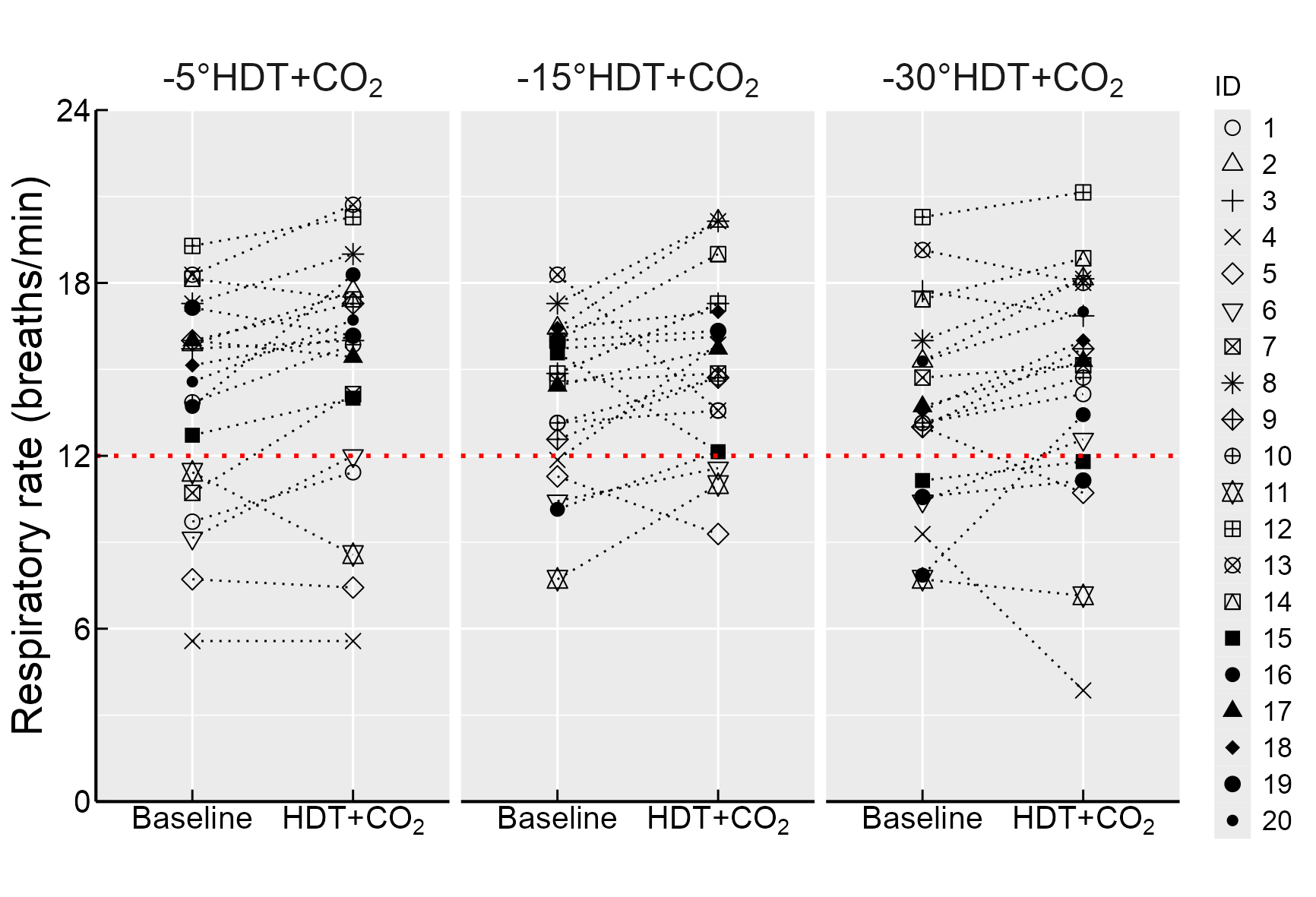


| **Supplementary file 4. The percentages of participants with a respiratory rate < 12 breaths/min** | | | | | | | | |  |
| --- | --- | --- | --- | --- | --- | --- | --- | --- | --- |
|  | -5°HDT+CO_2_ | |  | -15°HDT+CO_2_ | |  | -30°HDT+CO_2_ | |  |
|  | Baseline | HDT+CO_2_ |  | Baseline | HDT+CO_2_ |  | Baseline | HDT+CO_2_ |  |
| Resp-R < 12 breaths/min | 30% (6/20) | 20% (4/20) |  | 25% (5/20) | 15% (3/20) |  | 30% (6/20) | 25% (5/20) |  |
| HDT, head-down tilt; Resp-R, respiratory rate. | | | | | | | | |  |
|  |  |  |  |  |  |  |  |  |  |
